# Motivational context and neurocomputation of stop expectation moderate early attention responses supporting proactive inhibitory control

**DOI:** 10.3389/fnhum.2024.1357868

**Published:** 2024-04-02

**Authors:** Resh S. Gupta, Alan N. Simmons, Nathalie N. Dugas, Daniel M. Stout, Katia M. Harlé

**Affiliations:** ^1^Department of Psychological & Brain Sciences, Washington University in St. Louis, St. Louis, MO, United States; ^2^Center of Excellence for Stress and Mental Health, VA San Diego Healthcare System, San Diego, CA, United States; ^3^Department of Psychiatry, University of California, San Diego, La Jolla, CA, United States

**Keywords:** attention, inhibitory control, motivation, proactive control, stop signal task, reward, punishment, event-related potentials

## Abstract

Alterations in attention to cues signaling the need for inhibitory control play a significant role in a wide range of psychopathology. However, the degree to which motivational and attentional factors shape the neurocomputations of proactive inhibitory control remains poorly understood. The present study investigated how variation in monetary incentive valence and stake modulate the neurocomputational signatures of proactive inhibitory control. Adults (*N* = 46) completed a Stop-Signal Task (SST) with concurrent EEG recording under four conditions associated with stop performance feedback: low and high punishment (following unsuccessful stops) and low and high reward (following successful stops). A Bayesian learning model was used to infer individual's probabilistic expectations of the need to stop on each trial: P(stop). Linear mixed effects models were used to examine whether interactions between motivational valence, stake, and P(stop) parameters predicted P1 and N1 attention-related event-related potentials (ERPs) time-locked to the go-onset stimulus. We found that P1 amplitudes increased at higher levels of P(stop) in punished but not rewarded conditions, although P1 amplitude differences between punished and rewarded blocks were maximal on trials when the need to inhibit was least expected. N1 amplitudes were positively related to P(stop) in the high punishment condition (low N1 amplitude), but negatively related to P(stop) in the high reward condition (high N1 amplitude). Critically, high P(stop)-related N1 amplitude to the go-stimulus predicted behavioral stop success during the high reward block, providing evidence for the role of motivationally relevant context and inhibitory control expectations in modulating the proactive allocation of attentional resources that affect inhibitory control. These findings provide novel insights into the neurocomputational mechanisms underlying proactive inhibitory control under valence-dependent motivational contexts, setting the stage for developing motivation-based interventions that boost inhibitory control.

## 1 Introduction

Abnormalities in inhibitory control are present across a wide range of psychopathology (Robbins et al., 2012; American Psychiatric Association, 2013), including substance use and addictive disorders (Smith et al., 2014), obsessive-compulsive disorder (Mancini et al., 2018), autism spectrum disorder (Schmitt et al., 2018), anorexia nervosa (Bartholdy et al., 2017), and schizophrenia (Zandbelt et al., 2011). Notably, these deficits often predict worsening of psychiatric symptoms and generally poorer clinical outcomes, including increased rates of intrusive maladaptive thoughts (Craske et al., 2008; Aupperle et al., 2012), self-harm, substance abuse, and other risky behaviors (Robbins et al., 2012; American Psychiatric Association, 2013), underscoring the need to understand the neural mechanisms and factors that modulate inhibitory control success.

One promising target for modulation is proactive inhibitory control, or the adaptive preparatory process that regulates the potential of inhibition success in the near future (Van Den Wildenberg et al., 2022). Indeed, this aspect of inhibitory control optimizes inhibitory performance by maintaining learned, top-down goal-relevant information; this can, in turn, guide early attention and action processes rather than relying on a reactive remediation process to abort the implementation of an unwanted automatic response (Braver, 2012). Importantly, motivational factors can influence inhibitory performance goals by directly modulating attentional mechanisms deployed to process inhibitory context and detect cues signaling the need for inhibition (Meyer and Bucci, 2016). Yet, the degree to which proactive inhibitory control mechanisms are modulated by motivational factors (e.g., reward, punishment) remains poorly understood. Recent behavioral studies have shown that reward motivation can improve inhibitory control processes (Chiew and Braver, 2014; Herrera et al., 2014, 2019; Giuffrida et al., 2023), and, in line with modulation of proactive inhibitory control, reward incentives can enhance transient neural responses of early attention to go stimuli prior to inhibitory cues (Schevernels et al., 2015; Langford et al., 2016). However, these studies did not examine the impact of motivation on the predictive neurocognitive processes supporting inhibitory performance. For instance, does motivational context bias learning and prediction of inhibitory actions and/or early attention independently, or do both motivational context and prediction of the need to inhibit interact to modulate early attention?

Computational cognitive models have proven increasingly useful to answer these types of questions by providing detailed mechanistic insights into complex cognition, such as learning-based executive processes (Botvinick and Cohen, 2014), and how these mechanisms are, in turn, impacted by affect, motivation, or psychopathology (Huys et al., 2016). Inhibitory control, learning, and prediction of inhibitory response needs have been well captured by the Dynamic Belief Model (DBM) (Yu and Cohen, 2008), a Bayesian-inference learning model applied to the stop-signal task (SST). The SST is a speeded choice reaction task in which one must respond to “go” stimuli and occasionally receive a stop signal (e.g., auditory tone) that cues them to withhold their response (Logan and Cowan, 1984). Using the formalism of probability distributions to represent expectations, the DBM model assumes that individuals dynamically update their beliefs about the likelihood of encountering a “stop” signal on a given trial (i.e., the “need to stop”) based on the cumulative history of trial type outcomes. This model further assumes that individuals adjust their behavior as a function of these expectations, specifically being more cautious and slowing down “go” reaction times when expectation of a stop signal is high. Thus, the DBM model offers a powerful quantitative account of proactive anticipatory processes supporting inhibitory performance by isolating dynamic predictions pertaining to inhibitory performance.

This Bayesian inference account of inhibitory control performance is aligned with predictive coding theory (Rao and Ballard, 1999) and the free energy principle (Friston, 2005, 2009) according to which any self-organizing system must minimize its free energy, i.e., unresolved uncertainty about its environment in order to maintain equilibrium and preserve its integrity (Friston, 2005). Within this hierarchical predictive coding framework, prior expectations about an upcoming stimulus, which we model here with DBM, act as top-down signals that are combined with sensory evidence via Bayes inference principles to minimize prediction error and improve active prediction of the bottom-up input. DBM modeling has the additional advantage of explaining behavioral adjustments to contextual manipulations (e.g., reward/punishment contingencies associated with performance, fluctuations in true stop signal frequency) (Shenoy and Yu, 2011). For instance, previous work has shown that DBM outperforms standard error-correction models (e.g., Rescorla-Wagner) to capture individuals' reward-based decisions (Harlé et al., 2015b, 2017), with both higher negative and lower positive affect relating to increased model-based expectation of reward volatility and reduced reward maximization (Harlé et al., 2017). Therefore, DBM is well suited to assess how motivational context impacts individuals' subjective expectations of the need to inhibit an automatic response and whether these anticipatory processes interact to shape proactive control of attention.

Prior neural investigation of DBM-based stop expectations have mostly relied on fMRI, with evidence of expectation-weighted activation to Go stimulus onset within the superior, medial inferior frontal regions, and the inferior parietal lobule, as well as in some studies within the parahippocampal and occipital cortex (Ide et al., 2013; Harlé et al., 2014, 2016, 2019; Hu et al., 2015a,b, 2016). However, the pace of the SST paradigm and the limited temporal resolution of fMRI can make it difficult to gage how tonic, top-down anticipatory predictions (more likely to be effectively captured by fMRI) modulate phasic responses during early sensory processing stages of the Go stimulus (e.g., bottom-up visual association cortices). To address this caveat and capitalize on the precision of trial-level model-based predictions, event-related potentials (ERPs) derived from the electroencephalogram (EEG) are a critical tool to measure early attention deployment. ERPs provide the temporal resolution necessary to identify proactive neural responses and transient modulations of attention preceding stop signals and prior to the implementation of response inhibition (Schevernels et al., 2015). Within the hierarchical Bayesian brain framework highlighted above (Friston, 2005, 2009), we predict that DBM-based expectations would exert top-down control on sensory processing areas such as the visual association cortices, as observed in some fMRI studies of DBM modulation in the SST (Hu et al., 2015b, 2016). The P1 and N1 visual ERP components are particularly well-suited for examining proactive allocation of attention supporting inhibitory control. The P1 and N1 components are generated in the extrastriate cortex, are modulated by attention, and are thought to reflect “gain control” of sensory processing (Luck et al., 2000; Finnigan et al., 2011). In cognitive paradigms, the P1 may reflect cognitive modulation processes, such as engagement in attention necessary for successful cognitive modulation of sensory processing (Kaiser et al., 2020). Additionally, N1 amplitudes are larger in a stop signal context compared to an ignore signal context, demonstrating that processing of the go stimulus is influenced by anticipation of a stop signal (Elchlepp et al., 2016). Similarly, being prepared to stop results in enhanced attention for relevant visual signals, reflected in an increased target-evoked visual N1 (Liebrand et al., 2017).

Thus, in this study, we investigated the role of expectation and motivation on neural measures of proactive inhibitory control. We used a motivated SST with concurrent EEG recording, in which participants are presented with, in separate blocks, specific types of feedback based on the ability to withhold their response to the stop-signal stimulus. Each block was associated with a unique motivational context, with low and high levels of monetary reward (for successful stops) and punishment (for unsuccessful stops). Our aims were threefold. First, we sought to assess whether motivational context associated with stop performance in the SST modulates (a) predictive neurocomputational processes supporting proactive inhibitory control, and/or (b) early attention processes in anticipation of inhibitory cues. Second, we examined whether inhibitory predictions influence early attentional P1/N1 responses independently or as a function of motivational context. Third, we investigated whether interactions between motivational contexts, inhibitory predictions, and early attentional P1/N1 predict inhibitory control success.

## 2 Materials and methods

### 2.1 Participants and procedure

Healthy adults from the community were recruited through ads and flyers posted around the University of California San Diego campus, as well as through ResearchMatch. Adults had to be between 18–55 years old and have normal or corrected vision and hearing to be included in the study. Participants were excluded based on report of past or current severe mental illness (i.e., mania, psychosis, severe substance use disorder).

**Figure 1:**
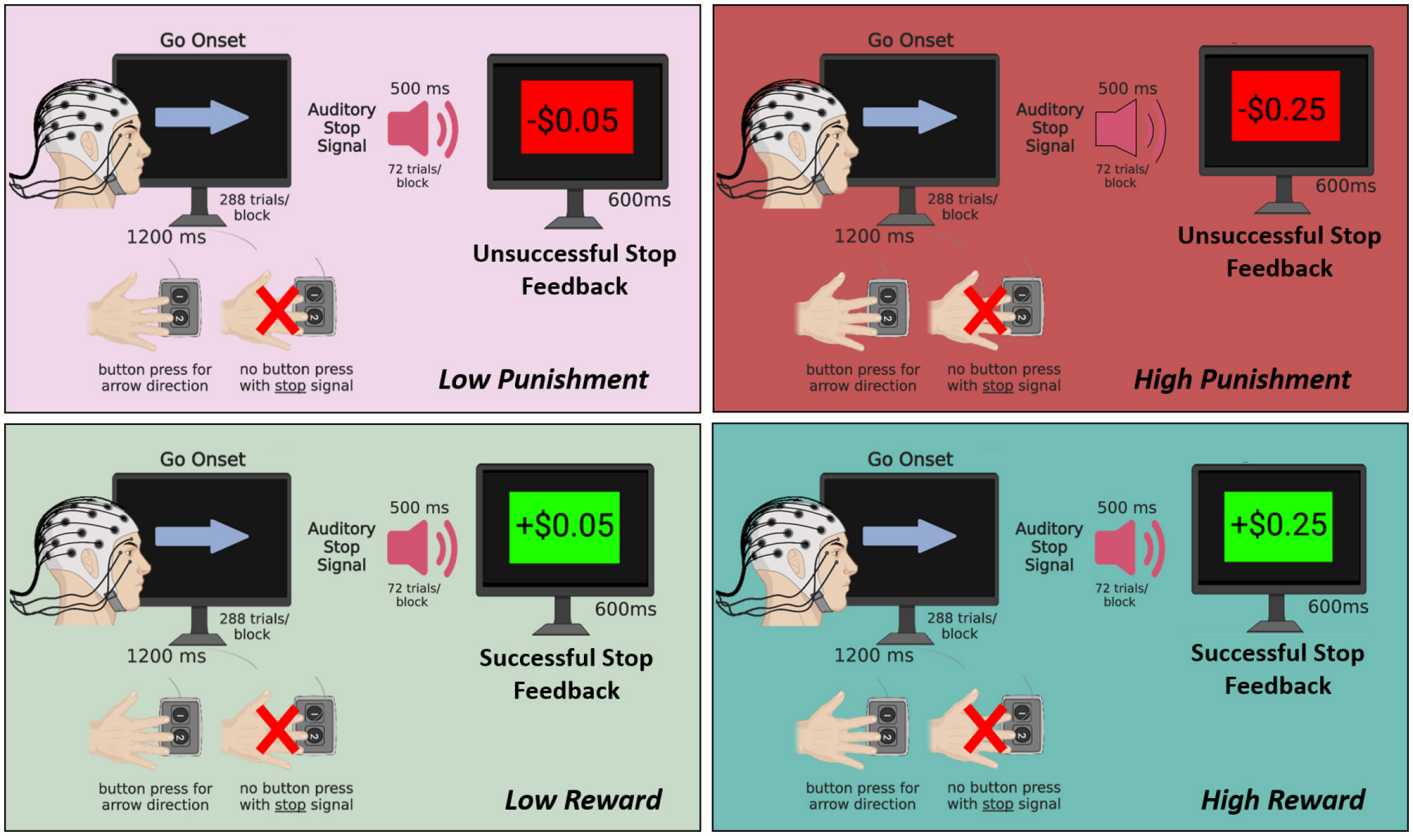
Schematic of the motivated SST. Participants completed 4 blocks of 288 trials each with 1 block for each of the following conditions: high reward (+$0.25 per successful stop), low reward (+$0.05 per successful stop), low punishment (–$0.05 per unsuccessful stop), and high punishment (–$0.25 per unsuccessful stop). In all blocks, a go-onset stimulus (i.e., arrow) was presented for 1,200 ms, and participants were required to press a button to indicate the arrow direction as quickly as possible. On 25% of trials, an auditory stop signal (500 ms duration) presented shortly after the arrow indicated that participants should withhold their button press to the arrow. Subsequently, a stop feedback screen was presented for 600 ms reflecting the monetary gain or loss depending on the condition. Figure created using BioRender.com.

Forty-nine participants completed the motivated SST with concurrent EEG recording under conditions of high punishment (HP), high reward (HR), low punishment (LP), and low reward (LR) (see Figure 1). Data from 46 participants (25 females) was analyzed, including 34 participants retaining all 4 conditions and 12 participants missing 1–3 conditions (HP: 91.30% retained; HR: 91.30% retained; LP: 84.78% retained; LR: 91.30% retained). Three subjects were dropped from the analyses due to noisy EEG data in all four SST conditions. Participants' mean age was 28.76 years (*SD* = 10.81). In terms of ethnicity, 13.04% were Hispanic/Latino, and in terms of race, 32.61% were Asian, 13.04% were Black/African American, 41.30% were White/Caucasian, 10.87% were more than one race, and 2.17% were unknown/not reported.

#### 2.1.1 Ethics statement

All participants provided written informed consent and received monetary compensation for their participation. The study was approved by the University of California San Diego Institutional Review Board.

### 2.2 Measures

#### 2.2.1 Motivated stop-signal task

A motivated SST with simultaneous EEG recording was used to assess how high and low levels of monetary reward and punishment modulate ERPs associated with proactive allocation of attention supporting inhibitory control (see Figure 1). Participants completed, in randomized order, 4 SST blocks of 288 trials each with 1 block for each of the following conditions: HR (+$0.25 per successful stop), LR (+$0.05 per successful stop), LP (–$0.05 per unsuccessful stop), and HP (–$0.25 per unsuccessful stop). In all blocks, a go-onset stimulus (i.e., arrow) was presented for 1,200 ms, and participants were required to press a button to indicate the arrow direction as quickly as possible. On 25% of trials (*n* = 72), an auditory stop signal (500 ms duration) presented shortly after the arrow indicated that participants should withhold their button press to the arrow. Subsequently, a stop feedback screen was presented for 600 ms reflecting the monetary gain or loss depending on the condition. Trial type order (go vs. stop trial) was randomized based on the fixed 75%/25% non-stop vs. stop trial contingency. Half of the trials had left-pointing arrows and half had right-pointing arrows, which appeared in randomized order. A 400 ms inter-stimulus interval (ISI) was included between the end of feedback presentation and next Go stimulus onset. The task used a staircase adaptive procedure, with task difficulty (i.e., stop signal delays/SSD) being adjusted in 50 ms up- or downward increments based on tracked performance to target a 50% stop error rate (Verbruggen et al., 2019). SSD was initialized at 500 ms with minimum and maximum boundaries set at 50 ms and 1,200 ms, respectively. Participants first completed a practice block and were required to meet 80% button press accuracy to receive earnings for a given block. In addition to their standard participation fee, all participants were paid in cash up to $25 based on their actual earnings in all SST conditions (not including the practice block) and were explicitly told this would be the case at the start of the experimental session.

### 2.3 Data analyses

#### 2.3.1 EEG data collection and processing

EEG was recorded continuously using Brain Vision Recorder (Brain Products GmbH, Gilching, Germany), actiCHamp Plus (Brain Products GmbH, Gilching, Germany), and a 64-channel actiCAP (Brain Products GmbH, Gilching, Germany) with a sampling rate of 500 Hz and referenced to the left mastoid. Vertical eye movements were recorded using electrooculogram (EOG) electrodes placed above and below the left eye, and horizontal eye movements were recorded using EOG electrodes placed near the outer canthus of each eye. Impedance of all channels was kept below 30 kΩ. Data were processed using Brain Vision Analyzer (Brain Products GmbH, Germany). Data were first filtered between 0.1 and 30 Hz via zero-phase shift band-pass (IIR Butterworth) filters. Raw data inspection was performed on the continuous EEG data to identify and mark artifacts. Ocular artifacts were corrected using the regression method (Gratton et al., 1983). Data were subsequently re-referenced offline to the average of the left and right mastoids. Topographic interpolation by spherical splines was performed on channels where >30% of trials were bad. Within all blocks (HP, HR, LP, LR), data were segmented from −200 ms before to 800 ms after go onset stimulus presentation. Artifact rejection was completed using automatic inspection, individual channel mode, and the following criteria: Maximal allowed voltage step: 50 μV/ms; Maximal allowed difference of values in intervals: 80 μV. Segments were baseline corrected using a window of −200 to 0 ms.

Primary ERP analyses focused on P1 and N1 amplitudes elicited by the go-onset stimulus at the level of single trials. P1 and N1 amplitudes were examined at electrodes PO7 and PO8 based on methods used in a rewarded stop-signal task ERP study (Schevernels et al., 2015). Subsequently, we used a collapsed localizer approach at electrodes PO7 and PO8 to identify time-windows for each component (Luck and Gaspelin, 2017). A 90–120 ms search window at electrodes PO7 and PO8 was used to identify P1 peaks in each trial, and a 130–180 ms search window at PO7 and PO8 was used to identify N1 peaks in each trial. The mean amplitude around the peaks at PO7 and PO8 were extracted (−24 ms before to 24 ms after the peak) for each trial, averaged to form a combined PO7/PO8 peak for each trial, and entered into model-based analysis described below.

#### 2.3.2 Computational modeling

As in previous work (Harlé et al., 2014, 2020), we used a dynamic Bayesian model (DBM) to estimate the prior expectation of encountering a stop signal on each upcoming trial, based on prior trial type history. This trial-level expectation is in turn used to adjust Go stimulus response times based on the likelihood of the need to stop this response. According to this model, individuals believe that stop signal frequency r_*k*_ on trial k has probability α of being the same as *r*_*k*−1_, and probability (1 – α) of being re-sampled from a fixed prior beta distribution *p*_0_*(*r_*k*_). Probability of a trial not having a stop signal (i.e., a “Go” trial) is assumed to be 1 – *r*_*k*_. Specifically, given the previous posterior distribution *p*(*r*__*k*_−1_ | **S**_*k*−1_) on trial k−1, where **S**_*k*−1_ is a vector from s_1_ to s_*k*−1_, representing all past trial outcomes up to k-1, the iterative prior distribution of stop signal frequency on trial k is given by:


$$\begin{array}{l} p\left(r_{k}\;|\;{\text{S}}_{k-1}\right)=\;\alpha \;p\left(\;r_{k-1}|\;{\text{S}}_{\;k-1}\right)+\left(1-\alpha \right)\;p_{0}\left(r_{k}\right) \end{array}$$

The prior distribution *p*_0_(r_*k*_) is assumed to be a beta distribution *Beta*(*a,b*), reparametrized with prior mean m = *a/(a*+*b)* and scale parameter *s* = (*a*+*b*). The new posterior distribution is then computed from the prior distribution and the observed outcome according to the Bayes' rule:


$$p(r_{k}\;|\;S_{k})\;\propto P(s_{k\;}|\;r_{k})\;p(r_{k}\;|\;S_{k-1})$$

The predicted probability that trial *k* is a stop trial, i.e., “P(stop),” can be expressed as *P*(s_*k*_ = 1|**S**_k − 1_), which is the mean of the predictive distribution *p*(r_*k*_ | **S**_*k*−1_):


$$\begin{array}{c} p\left(s_{k}=1|\;{\text{S}}_{k-1}\right)=\;\int P\left(s_{k}=1\;|{\;r}_{k}\right)p\left(r_{k}\;|{\;\text{S}}_{k-1}\right)dr_{k}\\ =\int r_{k}p\left(r_{k}\;|{\;\text{S}}_{k-1}\right)dr_{k}=\;\langle \;r_{k}\;|\;{\;\text{S}}_{k-1}\rangle \end{array}$$

Based on the model assumption of a positive linear relationship between expectation of a stop signal and Go stimulus reaction times (RTs), best fit parameters (α, *m, s*), and associated P(stop) sequence for each trial were estimated by maximizing the robust linear regression fit (*R*^2^) of P(stop) and all non-omission Go RT for each condition block of each participant (search spaces tested: α = [0.25, 0.26,…, 1.00], *m* = [0.01, 0.02,…, 0.99], *s* = [2, 4, …, 20]).

#### 2.3.3 Statistical analyses

Individual-level behavioral measures (e.g., SSRT; see Harlé et al., 2014; Berner et al., 2023 for computation) and best fit DBM model parameters were compared across motivation conditions with linear mixed-effect models (LME), testing main effects and interaction of condition valence (reward vs. punishment) and stake (low vs. high), treating subject as a random factor. To examine the impact of motivation on the modulation of reaction times by P(stop), we applied a LME to participants' Go RTs during non-stop trials, treating subject as a random factor and condition valence, stake, and trial-level P(stop) as fixed effects. A generalized LME with a logit link function was applied to binary stop error performance (0 = no button press; 1 = failed inhibition of button press) with the same predictor structure was used to investigate modulation of stop performance. Finally, two similar LMEs tested the main effects and interactions of condition valence (reward vs. punishment), condition stake (low vs. high), and trial-wise P(stop) on participants' trial-level standardized peak amplitude of parietal/occipital P1 and N1 ERPs, respectively (see above for ERP description). To ensure results from these analyses would not be confounded by stop signal processing related activity, trials with SSD ≤ 150 ms (*n* = 287 trials, 0.6% of total number of trials) were removed from LMEs involving trial-level ERP data. For binary dependent variables (e.g., trial-level stop and go error data), we applied a binomial distribution and logit link function in generalized LME analyses. To gage whether the modulating effects of incentive motivation on the neural markers of proactive control further extended to inhibitory performance, we included in two separate LMEs, trial-level P1 and N1 peak amplitude, respectively, as a 4^th^ predictor of stop error in addition to motivation valence, stake, and P(stop). We only highlight here significant effects and interactions involving P1 or N1 amplitude [see above for effects of P(stop) and its interaction with condition]. All mixed-linear model analyses were estimated within a Bayesian framework (Wagenmakers et al., 2018; Keysers et al., 2020), using the Rstan based package brms (Bürkner, 2017). Uniform *Beta*(1,1) were specified for model coefficient priors. Posterior median and median absolute deviation (MAD)-based 95% high-density/credible intervals (HDI) were used to provide a robust estimate of each model parameter.

## 3 Results

### 3.1 Impact of motivation on behavior and model parameters

#### 3.1.1 Model parameters

There were no credible effects or interactions of incentive valence and stake on individual-level model parameters, including α and fixed prior parameters m and s (95% HDI included 0; see Table 1). Thus, motivational context did not significantly impact individuals' perceived stability of the stop signal rate, nor did it influence initial (i.e., first trial) expectations about stop signal frequency (fixed prior mean; see Methods).

**Table 1:** Behavioral performance by motivation condition.

	**Low reward**	**High reward**	**Low punishment**	**High punishment**	**Significant condition differences**
DBM α	0.95 ± 0.09	0.94 ± 0.10	0.94 ± 0.15	0.92 ± 0.16	n.c.^a^
DBM *prior mean*	0.33 ± 0.35	0.32 ± 0.36	0.28 ± 0.34	0.37 ± 0.40	n.c.^a^
DBM *prior scale*	14.26 ± 7.72	13.87 ± 7.59	15.91 ± 6.35	15.17 ± 7.04	n.c.
Mean Go RT (ms)	943 ± 188	954 ± 182	944 ± 187	953 ± 180	High>Low^*^
Go Success (%)	96.4 ± 18.7	96.8 ± 17.6	96.7 ± 17.8	96.7 ± 17.7	n.c.^b^
Stop Success (%)	54.2 ± 49.8	53.6 ± 49.9	53.9 ± 49.9	52.8 ± 49.9	n.c.^b^
SSRT (ms)	297 ± 56	300 ± 56	311 ± 41	312 ± 52	Punishment > Reward^*^

Values displayed are condition-level mean ± SD (*N* = 46); DBM, Dynamic Belief Model; SSRT, Stop Signal Reaction Time; ^a^Box Cox transformation was applied given non-normal distribution of this parameter; ^b^data analyzed with a generalized linear mixed effect (LME) with a binomial distribution applied to trial-level binary data and logit link function; n.c. indicates condition-related contrast not a credible effect (95% HDI included 0); ^*^indicate a credible effect or interaction of condition valence and/or stake (95% HDI does not include 0); HDI, High Density Interval.

#### 3.1.2 Go reaction times

**Figure 2:**
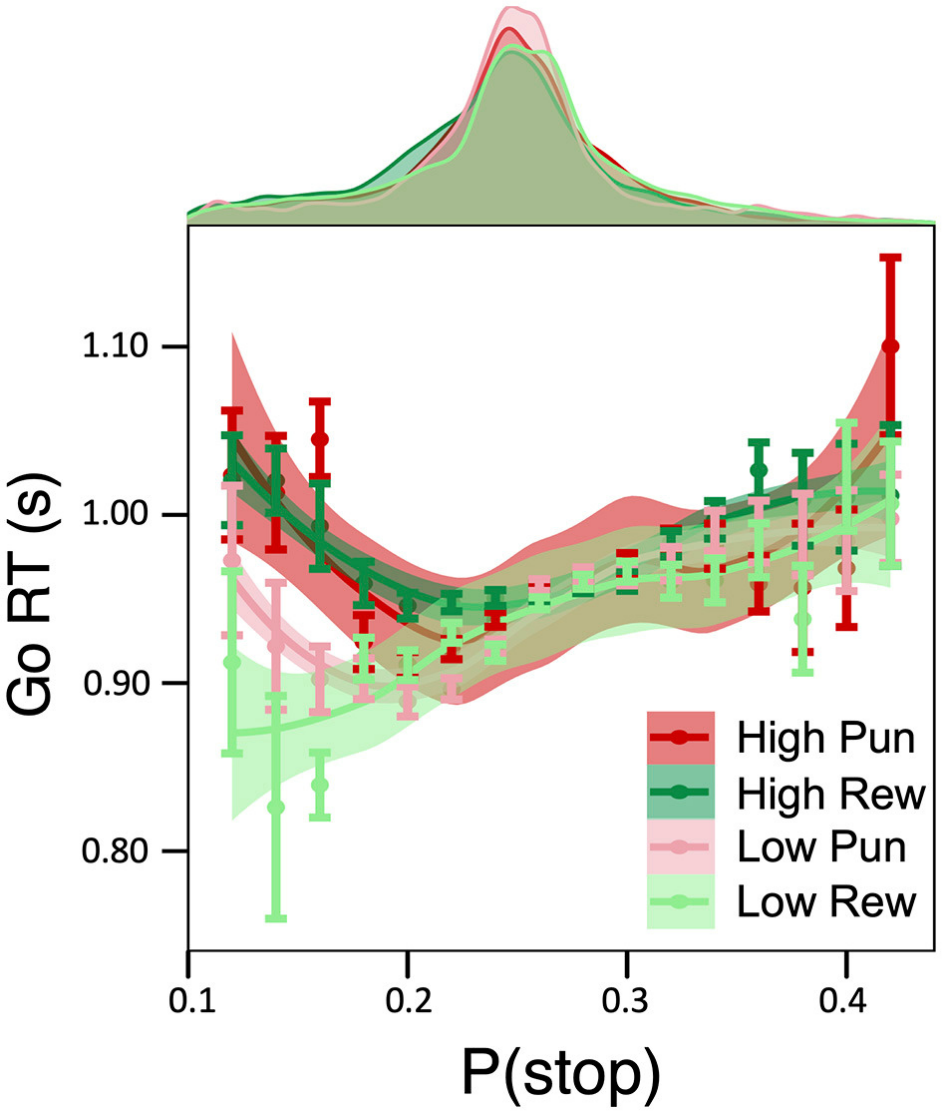
Effect of motivation on model-based inhibitory control. Relationship between Go RTs and P(stop) as a function of motivation condition: a Stake × P(stop) interaction was observed such that, for trials with low P(stop)/low expectations of a stop signal, a P(stop) was negatively related to RTs in high reward and high punishment conditions, which was less pronounced for low stake conditions.

A main effect of condition stake, but not valence, on Go RT was observed (β = 0.028, 95% HDI = [0.019, 0.036], 99.9% of posterior > 0), pointing to slower Go RTs in high relative to low stake (see Table 1 for condition-specific mean RT as well as Go accuracy, which did not differ as a function of condition). As predicted by DBM, a positive linear relationship between Go RTs and P(stop) was observed independently of motivation condition (β = 0.083; 95% HDI = [0.074, 0.091], 99.9% of posterior > 0; See Supplementary Figure S1). Importantly, these effects were qualified by a credible P(stop) × stake interaction (β = −0.022; 95% HDI = [−0.031, −0.014], 99.0% of posterior > 0), consistent with a stronger positive linear relationship between RT and P(stop) in low stake relative to high stake incentives. As can be seen in Figure 2, reaction time differences were most apparent for lower P(stop) values. No other credible main effect nor interactions were observed.

#### 3.1.3 Stop performance

Individuals' stop signal reaction times (SSRTs) differed based on condition valence. Specifically, longer SSRTs were observed in the punishment conditions (M = 312 ms) relative to the reward conditions (M = 299 ms; β = 0.14, 95% HDI = [0.01, 0.29], 97.7% of posterior > 0; see Table 1). Neither condition valence nor stake significantly directly predicted stop error (see Table 1 for error rates within each condition). Participants had a lower likelihood of error on trials with higher P(stop) values (odd ratio = 0.95; 95% HDI = [0.921, 0.985], 99.7% of posterior < 0), in line with our model's assumption that slowing down when stop expectation are higher helps improve stop accuracy.

### 3.2 Modulation of ERP peak amplitude by motivation and model-based stop expectations

#### 3.2.1 Modulation of P1 amplitude

**Figure 3:**
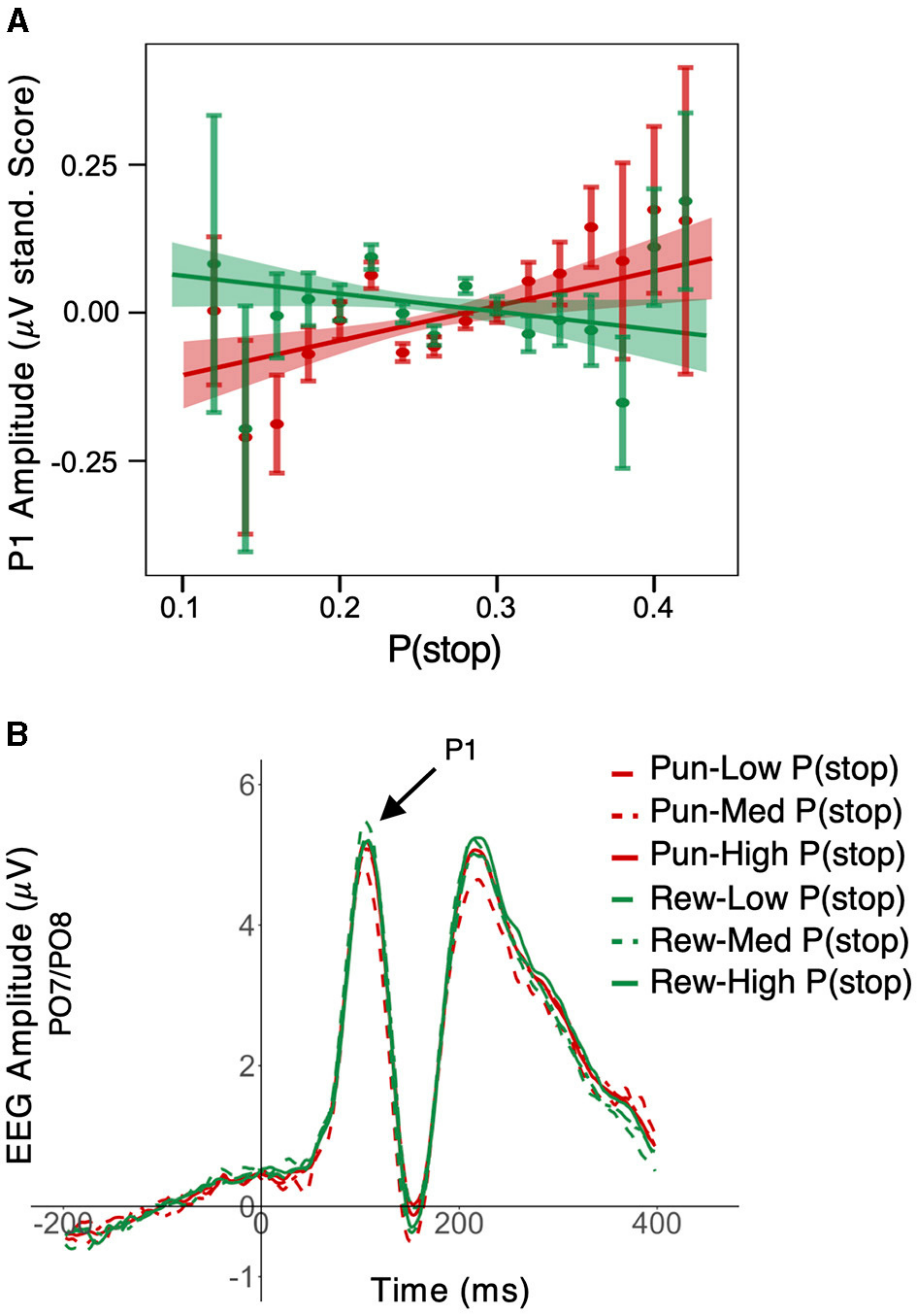
Effect of motivation and stop expectations on P1 ERP amplitude. **(A)** A Valence × P(stop) interaction was observed such that, trial-level P(stop) was positively related to P1 peak amplitudes in the punishment conditions, but not in the reward conditions; linear fit lines (with 95% Confidence Interval represented by shaded band); error bars represent SEM for binned P(stop). **(B)** For visualization purposes, we plotted average ERP timeseries as a function of motivation valence and P(stop) categories, i.e., low: P(stop) < 0.23, medium: 0.23 ≥ P(Stop) < 0.27, high: P(Stop) ≥ 0.27 (representing each tertile of the P(stop) distribution); P1 peak is situated in a 90–120 ms window (see arrow); P1 peak amplitude appears lower in the punishment relative to reward conditions for low P(stop)/low stop expectations trials; this pattern was less apparent for medium and high P(stop) trials.

A Bayesian LME model applied to standardized trial-level P1 peak amplitude revealed a significant main effect of condition valence (β = 0.008; 95% HDI = [0.001, 0.016], 97.8% of posterior > 0). This effect was qualified by a statistically significant P(stop) × valence interaction (β = −0.010; 95% HDI = [−0.018, −0.003], 99.4% of posterior < 0). Upon further examination P(stop) linear trends in each condition, P(stop) was positively related to P1 amplitude in punishment (β = 0.018; 95% HDI = [0.006, 0.030], 99.8% of posterior > 0) but not in reward conditions (β = −0.001; 95% HDI = [−0.011, 0.010], 51.1% of posterior < 0); see Figure 3A and see Figure 3B for average ERP timeseries as a function of condition and P(stop) level. For visualization purposes, we plotted average ERP timeseries as a function of motivation valence and P(stop) categories, i.e., low: P(stop) < 0.23, medium: 0.23 ≥ P(Stop) < 0.27, high: P(Stop) ≥ 0.27 [representing each tertile of the P(stop) distribution)]. See Supplementary Figure S2, top panel, for P1 scalp topographies across conditions.

#### 3.2.2 Modulation of N1 amplitude

**Figure 4:**
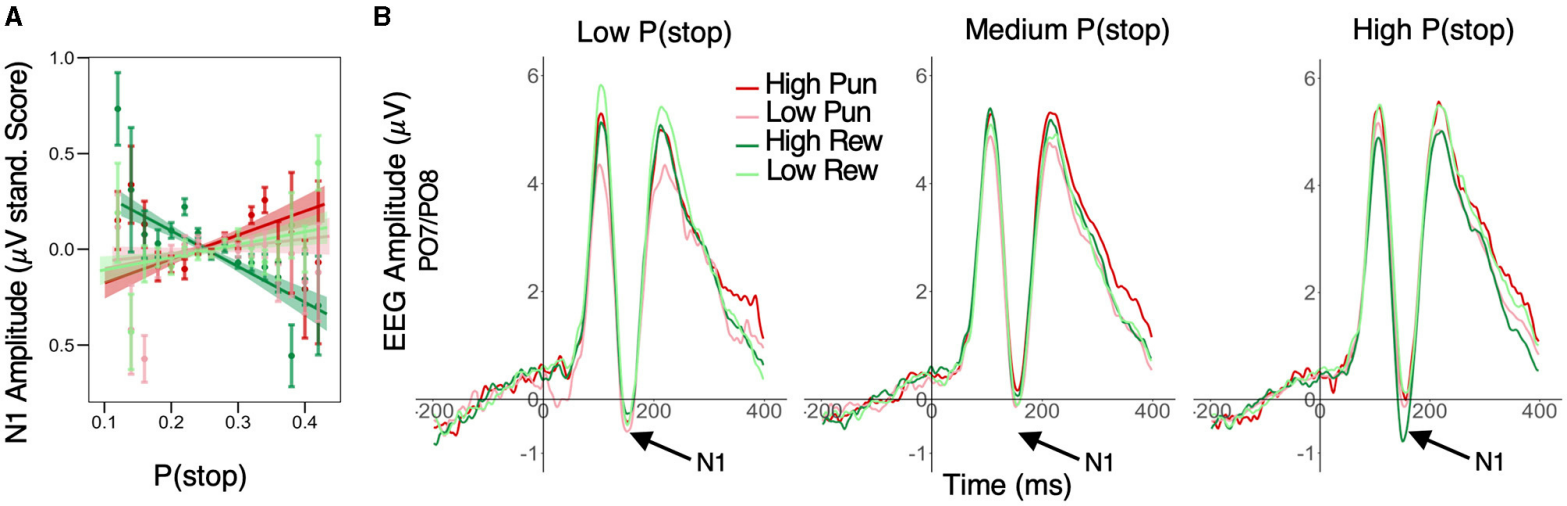
Effect of motivation and stop expectations on N1 ERP amplitude. **(A)** Valence × Stake × P(stop) interaction predicting N1: P(stop) was negatively related to N1 amplitude in the high reward condition, but positively related to N1 in the high punishment condition; linear fit lines (with 95% Confidence Interval represented by shaded band); error bars represent SEM for binned P(stop). **(B)** For visualization purposes, average ERP timeseries was plotted as a function of motivation and P(stop) categories, i.e., low: P(stop) < 0.23, medium: 0.23 ≥ P(Stop) < 0.27, high: P(Stop) ≥ 0.27 [representing each tertile of the P(stop) distribution]; N1 peaks are situated in a 130–180 ms window (see arrows); N1 amplitude was more negative in high reward condition for high P(stop)/high stop expectations trials; this pattern was less apparent for low and medium P(stop) trials.

A similar LME model was applied to standardized trial-level N1 peak amplitude. This analysis first revealed significant main effects of condition valence (β = 0.011; 95% HDI = [0.004, 0.019], 99.7% of posterior > 0) and stake (β = 0.022; 95% HDI = [0.014, 0.030], 99.9% of posterior > 0), with greater negative amplitudes in punishment relative to reward conditions and in low stake relative to high stake conditions, respectively. Importantly, these condition effects were qualified by a significant P(stop) × valence × stake interaction (β = −0.012; 95% HDI = [−0.020, −0.004], 99.8% of posterior < 0). More specifically, P(stop) was positively related to N1 amplitude in the high punishment (β = 0.019; 95% HDI = [0.002, 0.036], 98.5% of posterior > 0) but was negatively related to N1 amplitude (i.e., larger N1) in the high reward condition (β = −0.016; 95% HDI = [−0.032, −0.001], 97.5% of posterior < 0). However, P(stop) did not credibly predict N1 amplitude in low stake conditions (95% HDI included 0; see Figure 4A and see Figure 4B for average ERP timeseries as a function of condition and P(stop) category. For visualization purposes, average ERP timeseries was plotted as a function of motivation and P(stop) categories, i.e., low: P(stop) < 0.23, medium: 0.23 ≥ P(Stop) < 0.27, high: P(Stop) ≥ 0.27 [representing each tertile of the P(stop) distribution)]. See Supplementary Figure S2, bottom panel, for N1 scalp topographies across conditions.

### 3.3 Effect of P1/N1 ERPs on stop performance and its modulation by motivational context and stop expectancy

#### 3.3.1 P1 amplitude

On stop trials, P1 peak amplitude did not significantly predict the odds of making stop error (i.e., failing to withhold response to Go stimulus; odd ratio = 0.99; HDI = [0.95, 1.03], 72.2% of posterior < 0), nor did it interact with valence, stake, or P(stop) to predict stop error (*ps* > 0.05; see Supplementary Table S1 for full model summary).

#### 3.3.2 N1 amplitude

**Figure 5:**
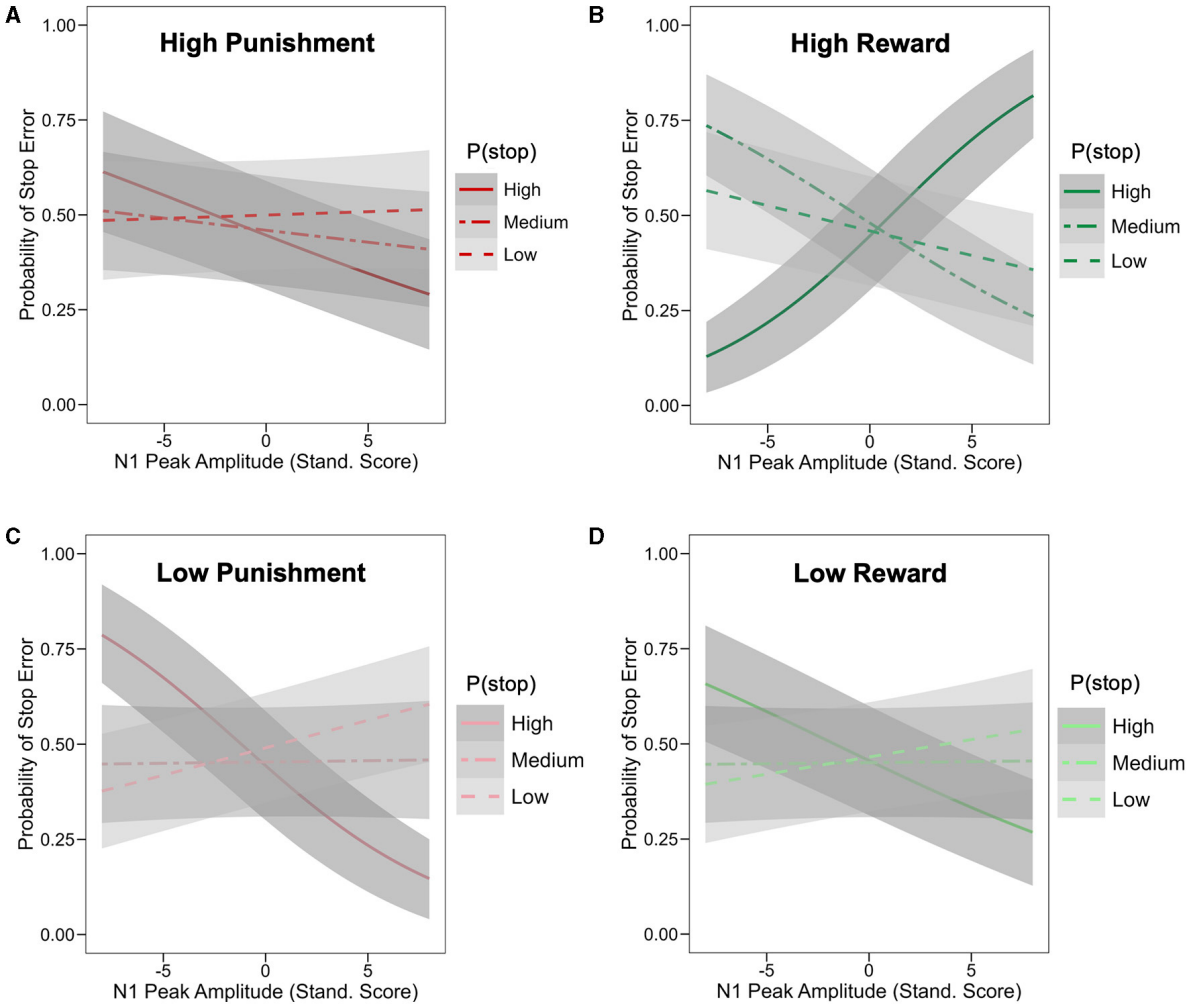
Effect of motivation, stop expectations, and N1 ERP amplitude on stop performance. Logistic model-based prediction of stop error as a function of N1 peak amplitude and P(stop) [based on tertile split: low: P(stop) < 0.23, medium: 0.23 ≥ P(Stop) < 0.27, high: P(Stop) ≥ 0.27]; each graph represents different motivation condition/block, including high punishment **(A)**, high reward **(B)**, low punishment **(C)**, and low reward **(D)**; shaded band represent 95% Confidence Interval; in the high reward condition **(B)**, more negative N1 amplitudes were associated with lower error likelihood for higher P(stop) values (odd ratio = 1.12; 95% HDI = [1.04,1.20]); the inverse pattern was observed in the low punishment (odd ratio = 0.91; HDI = [0.84,0.98]) and low reward (odd ratio = 0.92; HDI = [0.85,0.99]) conditions, in which more negative N1 amplitudes were associated with higher error likelihood for higher P(stop) values.

The main effect of N1 peak amplitude on stop trial error was not statistically significant (odd ratio = 0.98; HDI = [0.94, 1.02], 86.5% of posterior < 0). However, a significant 4-way interaction was observed between N1 amplitude, P(stop), valence and stake (odd ratio = 1.04; 95% HDI = [1.01,1.08], 98.0% of posterior > 0; see Supplementary Table S2 for full model summary). To unpack this interaction, we assessed, for each of the four motivation conditions (i.e., Low Punishment, High Punishment, Low Reward, and High Reward), the interaction of N1 amplitude and P(stop) predicting stop error. This interaction was not significant in the high punishment condition (odd ratio = 0.95; HDI = [0.88,1.02], 92.3% of posterior < 0; see Figure 5A [tertile split: low: P(stop) < 0.23, medium: 0.23 ≥ P(Stop) < 0.27, high: P(Stop) ≥ 0.27]. In the high reward condition, more negative N1 amplitudes were associated with lower error likelihood as P(stop) increased (odd ratio = 1.12; 95% HDI = [1.04,1.20], 99.9% of posterior > 0; see Figure 5B). The inverse pattern was observed in the low punishment (odd ratio = 0.91; HDI = [0.84,0.98], 99.2% of posterior < 0) and low reward (odd ratio = 0.92; HDI = [0.85,0.99], 98.8% of posterior < 0) conditions, in which more negative N1 amplitudes were associated with higher error likelihood as P(stop) increased (see Figures 5C, D). For visualization purposes, we also provide Johnson-Neyman plots of these 2-way interactions in each motivation condition (see Supplementary Figure S3), as well as violin plots of stop error rates as a function of N1 amplitude (i.e., small/less negative vs. large/more negative) and P(stop) category (by tertile; see Supplementary Figure S4).

## 4 Discussion

The present study investigated the role of motivation on neurocomputational markers of proactive inhibitory control. To do so, we assessed whether motivational context associated with stop performance in the SST (low or high reward/punishment) moderates anticipation of the need to stop as well as early attention neural responses (i.e., P1/N1 in parietal-occipital regions), and, critically, whether such potential biasing effects impact inhibitory performance. We observed three important and novel findings. First, motivational context interacted with model-based expectations at the trial level, i.e., P(stop), to modulate behavior, including proactive inhibitory control (Go reaction times) and reactive inhibitory control (stop signal reaction time, i.e., SSRT). Second, motivational context and P(stop) interacted to modulate P1 and N1 neural responses to the Go stimulus. Specifically, increasing levels of P(stop), i.e., higher expectation of the need to stop, in the punishment (but not reward) conditions was associated with greater P1 amplitudes. Moreover, higher P(stop) in the high-stake reward condition was associated with more negative N1 amplitudes, while the inverse pattern was seen in the other conditions, particularly the high-stake punishment condition. Third, in the high reward condition, increasing N1 amplitudes were associated with greater stop success when P(stop) was higher. These findings provide compelling evidence that subjective expectations to inhibit on a trial-to-trial basis modulate the effect of motivational context on neural measures of proactive control of attention. To our knowledge, the present study is the first to use DBM modeling to explore how monetary incentives interact with computationally driven anticipatory processes to modulate early attention ERPs associated with proactive allocation of attention supporting inhibitory control.

Previous studies in healthy populations (Ide et al., 2013) and in individuals with stimulant use disorder (Ide et al., 2015; Harlé et al., 2019), alcohol use disorder (Hu et al., 2015b), and eating disorders (Berner et al., 2023), have demonstrated that individuals slow down their Go reaction time in the SST as their expectation of the need to inhibit increases, according to a Bayesian inference anticipation process (Yu et al., 2009). Our findings add to this prior work as we found that, relative to low-stake conditions, high-stake motivation (high punishment and high reward) was associated with longer RTs primarily when P(stop) values were lower. Thus, while the typical positive linear relationship between P(Stop) and Go RT was observed for low stake conditions, high stakes of punishment or reward resulted in a dampening of this effect when expectation of a stop trial is low. One possible interpretation is that high stake monetary incentives associated with stop performance results in maintained cautiousness and slowing down in response to Go stimulus onset even when the need to inhibit the response is unlikely. We also found that reward motivation, i.e., wining money for successful inhibition, was associated with shorter stop signal reaction times (SSRTs). Overall, these results are consistent with prior findings that SST performance and measures of response inhibition are influenced by motivational context; participants respond more accurately on stop trials and respond more slowly overall as rewards and punishments increasingly favor stop accuracy over speed (Leotti and Wager, 2010). They are also consistent with findings that reward can enhance both proactive inhibitory control (slower Go RTs, physiological evidence of enhanced attention) (Chiew and Braver, 2014; Giuffrida et al., 2023) and reactive inhibitory control (lower SSRTs) (Herrera et al., 2019). However, we did not find any significant impact of motivation stake or valence on individuals' learning parameters that capture initial, pre-observation expectations and perceived stability of the stop signal frequency. Therefore, the present findings suggest that the influence of monetary incentives on inhibitory control may not be by biasing learning and development of stop expectations, but instead suggest that such motivational factors, particularly reward, may interact with top-down, model-based anticipatory processes to modulate inhibitory control performance.

Outcomes of prior experiences informs future expectations and directs attention to motivationally relevant features, particularly to cues predicting punishment (Suárez-Suárez et al., 2019). For example, early visual attention ERPs generated from the visual cortex are enhanced to stimuli associated with monetary loss relative to reward (Rossi et al., 2017). Consistent with this, we observed that in the punishment but not reward conditions, Go-related P1 amplitudes increased with higher levels of P(stop) expectations. The posterior visual P1 reflects engagement in attentional processing necessary for successful cognitive modulation of sensory processing (Kaiser et al., 2020) via the extrastriate cortex (Di Russo et al., 2002). This finding indicates that attentional enhancement to cues associated with punishment are modulated by an individual's expectations, particularly when there is a high expectation for the need to deploy inhibitory control when a failure to do so results in monetary loss. Punishment is a potent learning process that shapes behavior, and aberrations in neural circuits instantiating learning from negative consequences are thought to partially underlie internalizing and externalizing psychopathology (Jean-Richard-Dit-Bressel et al., 2018). Therefore, characterizing how punishments interact with cognitive features such as expectations and their underlying neural circuitry will be important for informing interventions that target these motivational processes (Mogg and Bradley, 2018).

Motivational incentives are key drivers of value-based attention (Anderson, 2016). Stimuli paired with high reward elicit larger visual attention ERPs originating from the visual and extrastriate cortices (i.e., P1 & N1) than stimuli paired with low reward (Hickey et al., 2010; MacLean and Giesbrecht, 2015; Schevernels et al., 2015). Midbrain and ventral striatal dopaminergic signaling bias early visual cortex to enhance attention to cues that have a history of being rewarded (Failing and Theeuwes, 2018; Anderson, 2019). Indeed, a positron emission tomography investigation showed that D_2_/D_3_ receptor signaling from the caudate when being rewarded predicted subsequent attention capture to stimuli that were previously rewarded (Anderson et al., 2017). Our findings build upon this literature showing that in the high reward condition, posterior N1 amplitudes to Go cues, an ERP associated with early visual attention (Boehler et al., 2009), were larger on trials with high P(stop) values relative to lower P(stop) values. Critically, this relationship predicted subsequent behavioral inhibitory control success. This pattern of findings suggests that trial-by-trial reward history, likely via striatal dopaminergic signaling (Anderson, 2019), informs or is informed by top-down proactive or expectancy signals likely emanating from the dorsolateral prefrontal cortex, pre-supplemental motor area, or the anterior cingulate cortex to guide inhibitory control (Watanabe, 2007; Harlé et al., 2014; Hu et al., 2016; Wang et al., 2018; Nobre and Stokes, 2019) enhances early visual attention to cues that signal the potential of obtaining a high-value reward. This early attentional enhancement subsequently facilitates the ability to inhibit motor responses in this high-stake reward context, even before the actual signal indicating the need to inhibit occurs (i.e., stop-signal). Collectively, these results provide compelling evidence that rewarded or motivationally relevant contexts interact with expectations to modulate proactive allocation of attention to support inhibitory control and maximize obtaining future rewards (Locke and Braver, 2008; Jimura et al., 2010).

The observed discrepancy between P1 and N1 findings are intriguing and may appear somewhat paradoxical. Specifically, P1 amplitude appears more broadly modulated by motivation valence and P(stop), whereas the level of stake plays an additional role in moderating N1 amplitude to in turn more robustly predict stop performance (which was not the case for P1 amplitude). These findings fit well with the hierarchical Bayesian nature of the brain predicted by the free-energy formulation of sensory learning and ERPs (Friston, 2005, 2009). In this framework, an ERP reflects the transient expression of free energy (i.e., prediction error), which is suppressed by top-down predictions from higher level cortical areas to varying degrees depending on prior learning. That is, those components may be quickly minimized if sensory input feedback matches expectations, or less so if the new information is novel or incongruent with the context (Friston, 2005). Through this lens, our findings would suggest that the P1 amplitude may be suppressed to a larger extent than N1, with N1 reflecting a different component of predictor error being unresolved and promoting subsequent action to resolve this unexplained discrepancy, hence more direct impact on behavior. Relatedly, and in line with the hierarchical organization of visual cortical layer and this Bayesian brain framework, the differential findings between P1 and N1 could reflect the temporal buildup of unexplained prediction error modulation on the Go stimulus onset processing. Later components may reflect Bayesian inference at more supraordinate level in the hierarchy, with longer latency responses reflecting increasing levels of Bayesian inference to integrate top-down contextual priors and bottom-up sensory input mediated by backwards connections (Friston, 2005, 2009; Garrido et al., 2007). Although P1/N1 are relatively early ERP components and we did not look at traditionally late components such as the P3 given its relevance to reactive vs. proactive cortical responses, our findings may point to the fact that N1 amplitudes are integrating a more relevant motivational context to trigger actions that minimize prediction error, i.e., fine-tuning stopping behavior to reduce sensorimotor surprise (Barceló and Cooper, 2018). This is reflected by the latter component (N1) having a richer, valence and stake- specific modulation and more direct influence on stop performance relative to P1.

The results of the current investigation demonstrate that motivational contexts and inhibitory expectations modulate neural markers of proactive control of attention and ultimately inhibitory control performance. Bayesian models offer a powerful, quantitative account of top-down cognition, such as inhibitory control, by isolating mechanistic markers of learning and anticipatory processes (e.g., dynamic predictions of the need to inhibit). This study combined such a cognitive computational modeling approach with high temporal resolution EEG methods to delineate where and how motivational factors are integrated into proactive inhibitory control. From a clinical perspective, our findings provide a roadmap for testing inhibitory deficits in psychopathology. First, leveraging computational modeling to understand inhibitory control deficits in psychopathology allows for the discovery of refined and nuanced relationships that non-computational analytic strategies may miss (Schall et al., 2017; Howlett et al., 2023). Indeed, DBM-based markers of inhibitory control show higher sensitivity and specificity in predicting clinical severity of substance use disorders (Harlé et al., 2015a, 2019; Hu et al., 2015b; Ide et al., 2015) and bulimia nervosa (Berner et al., 2023). Second, our results offer novel avenues for therapeutics that may improve inhibitory control. The ability to train and improve inhibitory control performance through cognitive training leveraging motivational incentives may be of great clinical relevance and a critical first step toward understating how such training effects can be transferred to intrinsic motivational factors (Jaeggi et al., 2014). It may also be fruitful to explore how motivational performance contingencies may enhance various therapeutic modalities, such as neurostimulation targeting early proactive control of attention (Pulopulos et al., 2022).

The present study is not without limitations. First, our sample consisted of adults with limited psychiatric exclusions and without formal diagnostic assessment. Therefore, we were unable to address whether findings are generalizable to individuals with or without psychiatric disorders. Future research recruiting both psychiatrically healthy and those with psychiatric disorders using clinician-administered diagnostic interviews are needed to explicate the role of psychopathology in these processes. Additionally, the present paradigm was not designed to assess feedback-related ERPs, which in turn limits our ability to capture how temporal and sensorimotor surprise, an important component of this inhibitory control task, further modulate sensory disambiguation of the Go stimulus, as predicted by Bayesian accounts of the brain (Friston, 2005; Friston et al., 2017). More specifically, the impact of prior stop trial prediction errors (e.g., in terms of SSD, but particularly stop outcome processing) may drive a significant portion of the P1/N1 modulation observed here, reflecting a hierarchy of sensorimotor loops (Garrido et al., 2007; Friston et al., 2017; Barceló and Cooper, 2018). There are practical reasons why we did not include neural responses pertaining to stop signals in the analyses, including the relative imbalance of Go vs. Stop trials (the latter being much fewer), the unattractable nature of the stop feedback processing (which includes both the auditory stop stimulus and the later explicit feedback), the absence of explicit feedback on non-Stop trials, and the relatively modest sample size, which further limits statistical power to tease apart the respective effect of these various types of surprise. Ideally, a more balanced design in which feedback trials are more frequent and feedback onset times can be reliably measured across both types of outcomes (e.g., go/nogo task, including explicit outcome type feedback on go trials) may be more suitable to investigate such complex dynamics. The use of information theory metrics (e.g., stimulus and response entropies) within a Bayesian framework may also be well suited to quantify additional contextual information predictive of ERP responses pertinent to proactive control (Friston et al., 2017; Barceló and Cooper, 2018). Ultimately, such an approach would be necessary to fully understand how the contextual feedback provided by value-based feedback drives individuals' dynamic adjustments of behavior in the SST. While it is not trivial to disentangle the dynamic interplay of these outcomes vs. sensorimotor surprise in the SST, our study provides a first window into understanding these mechanisms. Additionally, there may be neural sources other than the extrastriate cortex important for proactive inhibitory control. Given prior fMRI studies of DBM-based neuromodulation in the SST and evidence of P(stop)-weighted activation in frontoparietal regions, another interesting avenue for future studies will be to investigate more anterior modulations of the N1 within a potentially broader hierarchy of top-down influences on visual attentional processing. Finally, the present stop task included a relatively easy continuous performance task, which resulted in few Go errors (e.g., incongruent arrow direction/button press). An important next step would be to assess how additional sources of sensorimotor surprise stemming from heightened Go stimulus task difficulty further interact with motivational factors and stop expectations to shape early visual processing during proactive inhibitory control.

In conclusion, the present study demonstrates that the interaction between motivationally relevant contexts (i.e., different degrees of reward and punishment) and dynamic predictions supporting inhibitory control modulate proactive allocation of attentional resources to relevant visual features, which subsequently affects inhibitory control success. Findings from the present study contribute to a comprehensive understanding of the intricate interplay between motivational states, attention, and proactive inhibitory control. By integrating computational modeling and neural measures, these findings provide valuable insights into the mechanisms supporting inhibitory control and set the stage for illuminating how these mechanisms may be impaired in psychopathology, paving the way for more effective therapeutic targets.

## Data availability statement

The raw data supporting the conclusions of this article will be made available by the authors, without undue reservation.

## Ethics statement

The studies involving humans were approved by University of California San Diego Institutional Review Board. The studies were conducted in accordance with the local legislation and institutional requirements. The participants provided their written informed consent to participate in this study.

## Author contributions

RG: Data curation, Formal analysis, Visualization, Writing—original draft, Writing—review & editing. AS: Conceptualization, Data curation, Resources, Software, Writing—review & editing. ND: Data curation, Investigation, Writing—review & editing. DS: Conceptualization, Data curation, Formal analysis, Investigation, Methodology, Resources, Software, Supervision, Validation, Visualization, Writing—review & editing. KH: Conceptualization, Data curation, Formal analysis, Funding acquisition, Investigation, Methodology, Project administration, Resources, Software, Supervision, Validation, Visualization, Writing—original draft, Writing—review & editing.
