# Supplementary material for: Motivational context and neurocomputation of stop expectation moderate early attention responses supporting proactive inhibitory control
Source: Front Hum Neurosci. 2024 Apr 2;18:1357868. doi: 10.3389/fnhum.2024.1357868 (PMC11019005; doi:10.3389/fnhum.2024.1357868)
Supplement: Supplementary file 1 [file Data_Sheet_1.PDF]

Supplementary Material

Figure S1. Effect of Motivation on Stop Expectations

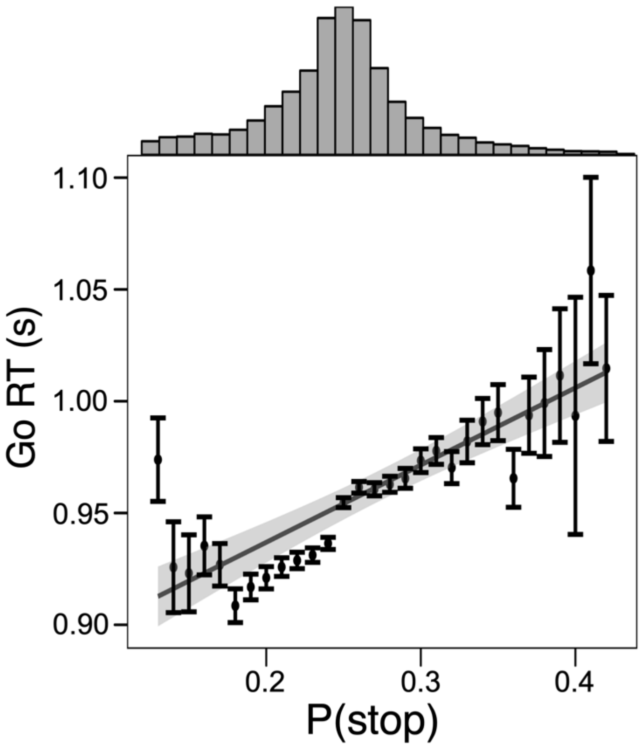

Note: Model fit across all condition blocks as predicted by the DBM model, a significant positive relationship was observed between individuals' Go Reaction Times (RTs) and their expectations of a stop signal, i.e., P(stop); black circles and error bars represent mean and SEM for each P(stop) bin.

Figure S2. Scalp topographies across conditions in the stop signal task.

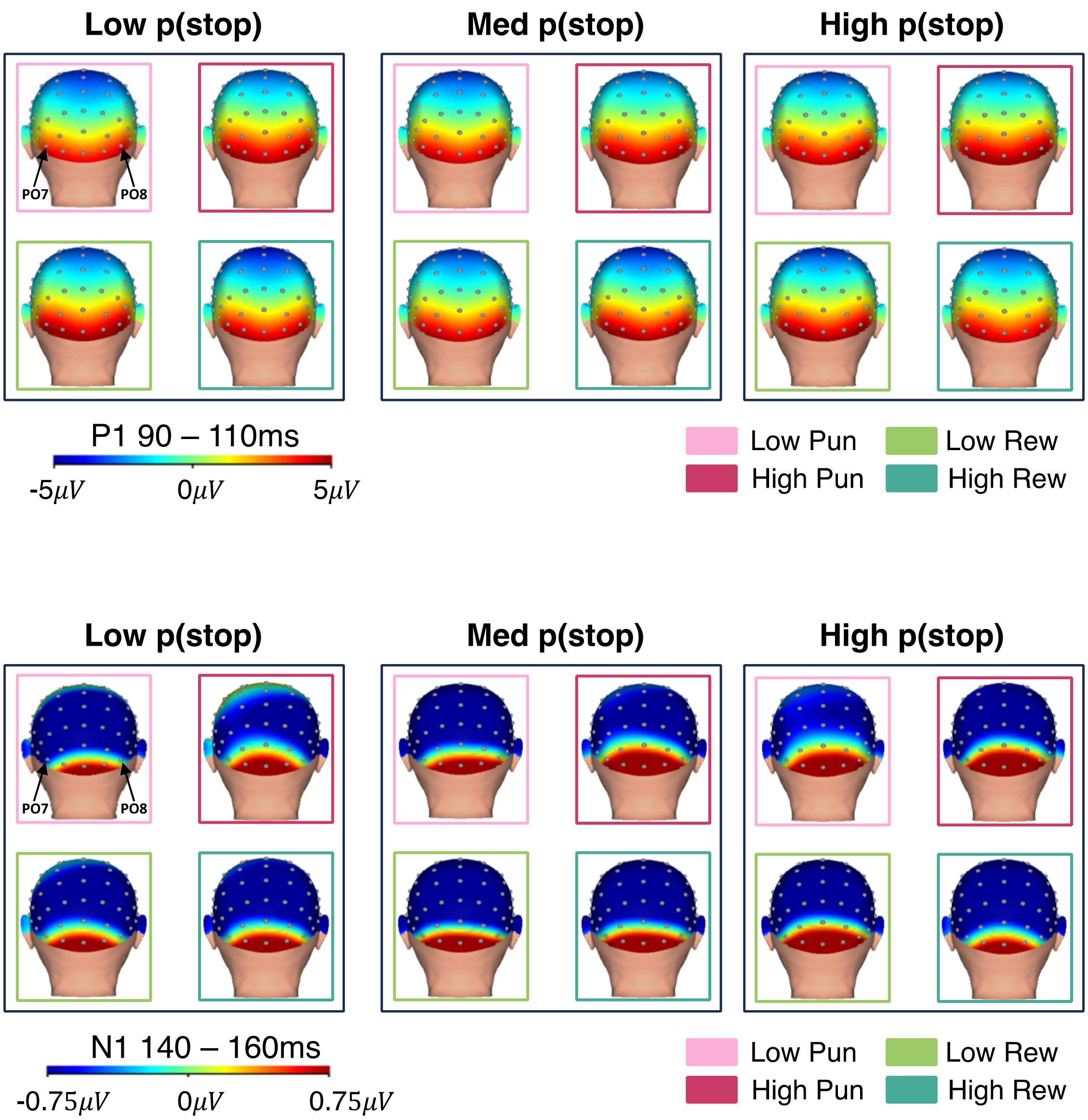

Note: P1 (top panel) and N1 (bottom panel) scalp topographies for low punishment (boxed in light pink), low reward (boxed in light green), high punishment (boxed in dark pink), and high reward (boxed in dark green) conditions within low, medium, and high p(stop) conditions. Data from electrodes PO7 and PO8, indicated with arrows in each panel, were analyzed.

Table S1. Linear mixed-effect model coefficients for the effect of P1 amplitude, P(stop), and motivation on stop error.

**Model Predictors**	**Bayesian Statistics**		**Frequentist Statistics**	
	**OR**	**95% HDI**	**$\chi^2(1)$**	***p***
P1	0.99	0.95 - 1.03	0.33	0.563
P(stop)	0.95	0.92 - 0.99	6.75	0.009
Val	0.99	0.95 - 1.03	0.45	0.502
Stake	1.01	0.97 - 1.05	0.34	0.558
P1*P(stop)	0.99	0.95 - 1.03	0.18	0.668
P1*Val	1.00	0.97 - 1.04	0.00	0.979
P(stop)*Val	1.03	1.00 - 1.07	3.07	0.080
P1*Stake	0.98	0.95 - 1.02	0.76	0.383
P(stop)*Stake	1.00	0.96 - 1.04	0.02	0.880
Val*Stake	1.00	0.97 - 1.04	0.04	0.845
P1*P(stop)*Val	1.02	0.98 - 1.06	1.20	0.273
P1*P(stop)*Stake	1.01	0.98 - 1.05	0.47	0.494
P1*Val*Stake	0.98	0.95 - 1.02	0.84	0.360
P(stop)*Val*Stake	1.00	0.96 - 1.04	0.01	0.922
P1*P(stop)*Val*Stake	1.02	0.99 - 1.06	1.46	0.227

Note: P1= P1 Peak Amplitude, Val=Condition Valence (Reward vs Punishment), Stake=Condition Stake (High vs Low), OR=Odds Ratio, HDI=High Density Interval.

Table S2. Linear mixed-effect model coefficients for the effect of N1 amplitude, P(stop), and motivation on stop error.

**Model Predictors**	**Bayesian Statistics**		**Frequentist Statistics**	
	**OR**	**95% HDI**	**$\chi^2(1)$**	***p***
N1	0.98	0.94 - 1.02	0.33	0.563
P(stop)	0.95	0.91 - 0.99	6.75	0.009
Val	0.99	0.96 - 1.03	0.45	0.502
Stake	1.01	0.98 - 1.05	0.34	0.558
N1*P(stop)	0.97	0.94 - 1.01	0.18	0.668
N1*Val	1.00	0.97 - 1.04	0.00	0.979
P(stop)*Val	1.04	1.00 - 1.08	3.07	0.080
N1*Stake	1.00	0.96 - 1.04	0.76	0.383
P(stop)*Stake	1.00	0.96 - 1.04	0.02	0.880
Val*Stake	1.00	0.97 - 1.04	0.04	0.845
N1*P(stop)*Val	1.05	1.01 - 1.09	1.20	0.273
N1*P(stop)*Stake	1.06	1.03 - 1.10	0.47	0.494
N1*Val*Stake	1.00	0.96 - 1.04	0.84	0.360
P(stop)*Val*Stake	1.00	0.96 - 1.04	0.01	0.922
N1*P(stop)*Val*Stake	1.04	1.00 - 1.08	1.46	0.227

Note: N1= N1 Peak Amplitude, Val=Condition Valence (Reward vs Punishment), Stake=Condition Stake (High vs Low), OR=Odds Ratio, HDI=High Density Interval.

Figure S3. Johnson-Neyman Plots for the interaction of N1 Peak Amplitude and P(Stop) on Stop Error Likelihood.

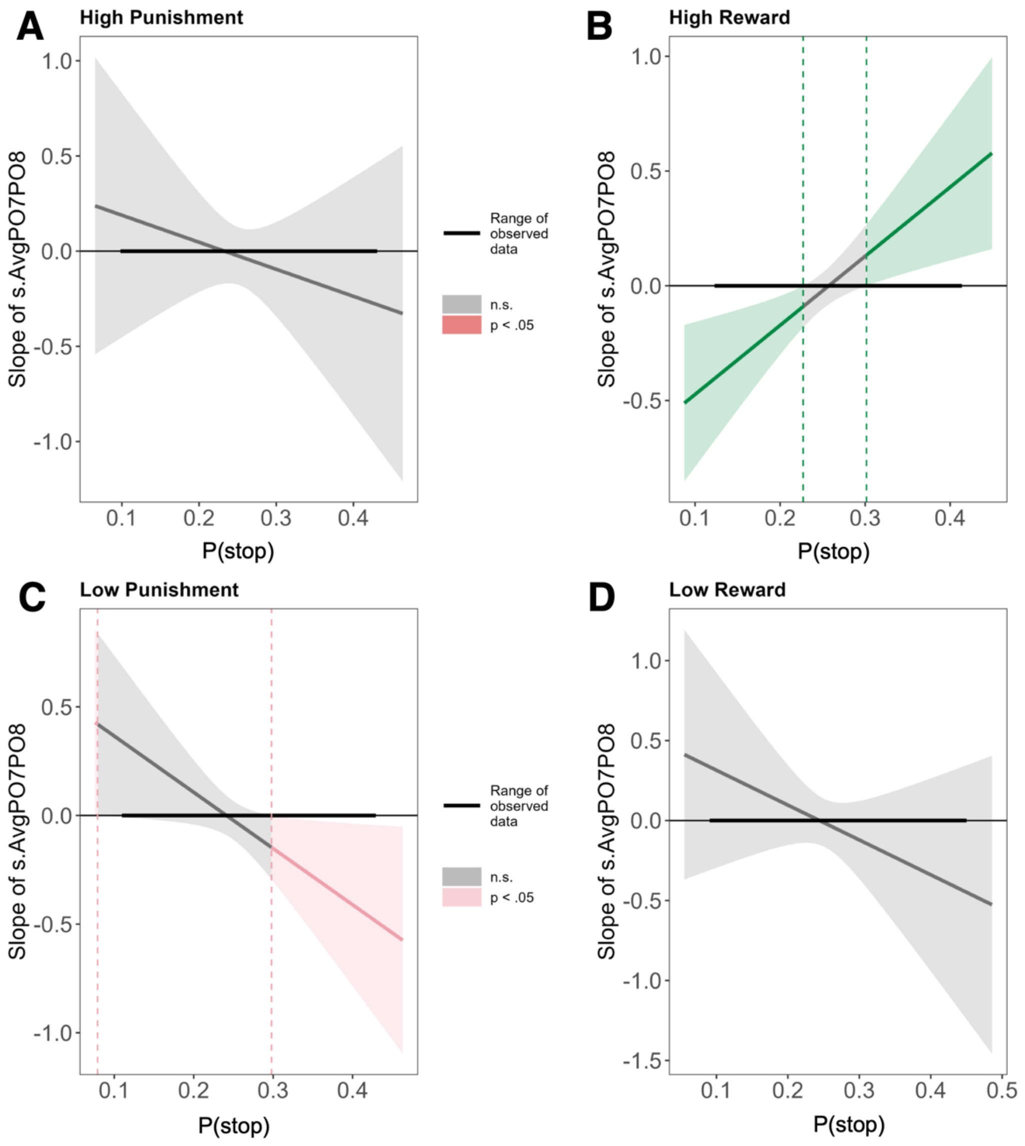

Note: Each plot shows the value range of moderator(P(stop) for which the conditional effect of N1 amplitude on Stop Error is significantly different from zero (grey areas) or significant (colored areas); shaded bands represent 95% Confidence Interval after FDR correction; within the range of observed data, P(stop) significantly moderated the effect of N1 on Stop error for values < 0.23 and > 0.30 in the High Reward condition (B), for values > 0.29 in the Low Reward condition (C).

Figure S4. Stop Error Rate as a function of Motivation, P(stop), and N1 ERP Amplitude.

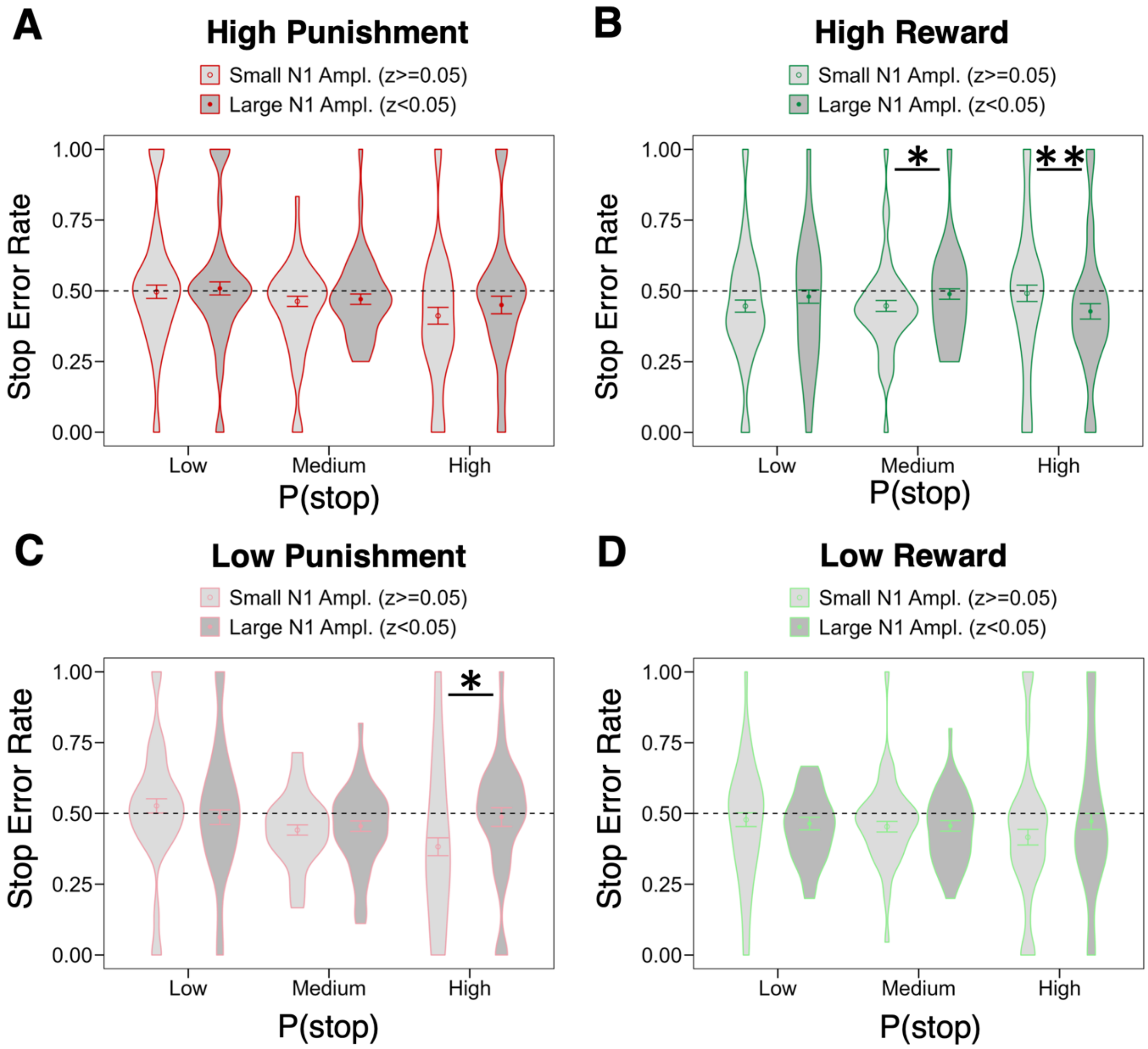

Note: In each graph, violin plots represent distributions of participants' stop error rates as a function of N1 amplitude (i.e., small/less negative vs large/more negative) and P(stop) categories (based on tertile split: low P(stop)<.23, medium: .23>=P(Stop)<.27, high: P(Stop)>=.27); each graph represents different motivation condition/block, including high punishment (A), high reward (B), low punishment (C), and low reward (D); circles and error bars represent group means and SEM respectively; *,**,*** indicate uncorrected statistically significant differences at $p<0.05$, $p<0.01$, $p<0.001$, respectively; only contrast for in high P(stop) of High Reward condition (FDR-adjusted p=0.048) survived FDR correction for 12 comparisons.
